# Cervical microbiota diversity and functional shifts in the development of cervical high-grade squamous intraepithelial lesions

**DOI:** 10.3389/fmed.2025.1615571

**Published:** 2025-10-24

**Authors:** Marta Rosas Cancio-Suárez, Elena Moreno, Cristina del Valle Rubido, Marta Salvador, Ana Moreno, Laura Luna, Claudio Díaz-García, Carlos Tapia, Ana del Amo, Santiago Moreno, Matilde Sánchez-Conde, Sergio Serrano-Villar

**Affiliations:** ^1^Department of Infectious Diseases, IRYCIS, and Universidad de Alcalá, Hospital Universitario Ramón y Cajal, Madrid, Spain; ^2^CIBERINFEC, Instituto de Salud Carlos III, Madrid, Spain; ^3^Department of Medicine, University of Alcalá de Henares, Guadalajara Campus, Alcalá de Henares, Spain; ^4^Department of Ginecology, IRYCIS, Hospital Universitario Ramón y Cajal, Madrid, Spain; ^5^Alpes Primary Care Center, Madrid, Spain; ^6^Department of Medicine, University Antonio de Nebrija, Life Sciences Campus, Madrid, Spain

**Keywords:** HSIL, cervicovaginal microbiota, 16S, HPV, bacterial functions

## Abstract

Research on microbial changes in the cervix, where most human papillomavirus (HPV) complications arise, is limited. Here, we aimed to understand the specific role of the cervicovaginal microbiota in developing high-grade squamous intraepithelial lesions (HSIL) associated with HPV infection. Our results show higher diversity in the microbiota associated with HSIL, with the genera *Parvimonas, Fastidiosipila*, and *Pseudomonas* being the most abundant. Additionally, an imputed functional analysis revealed that pathways such as glycine, serine, threonine, and sulfur metabolism were enriched in cervical samples from women with HSIL. Identifying biomarkers that help prevent HSIL progression could benefit women at risk of developing HPV-related cancerous lesions.

## Background

The cervicovaginal microbiota has co-evolved with mucosal immune cells to facilitate fetal implantation and development during pregnancy and protect the female urogenital tract from infection (1, 2). The correlation between cervicovaginal microbiota and its role in either fostering susceptibility or providing protection against HPV, the third leading cause of cervical cancer, is gaining attention (3).

Research on bacterial communities has revealed that the optimal vaginal microbiota is predominantly composed of *Lactobacillus crispatus*, *L. gasseri*, and *L. jenseni*. These species secrete lactobiocins and biosurfactants that prevent other species from binding to the epithelium (4). Interestingly, a vaginal microbiota dominated by *L. iners* has been associated with higher odds of HPV prevalence, including high-risk HPV and cervical cancer or dysplasia (5). It has been established that the interaction between HPV, host immunity, and the vaginal microenvironment influences the risk of persistent HPV infection and the progression of cervical intraepithelial neoplasia (CIN) (6, 7). However, while HPV-related complications primarily develop in the cervix, the cervical microbiota has been less studied. Comparative analyses not focused on HSIL further show cervix–vagina differences, underscoring site-specific composition and detectability, and reporting variable Lactobacillus dominance (8, 9). Furthermore, decreased levels of Lactobacillus have been reported in women with low HPV viral loads (10).

Therefore, we conducted a study to explore the connections between HPV infection and squamous intraepithelial lesions, focusing on the composition and function of the cervical microbiota. We aimed to identify metabolites that can help identify risk markers for malignant or premalignant lesions, enhance current screening efforts, and identify potential therapeutic targets in the long term.

## Methods

### Ethics

This study conformed to the principles of the Declaration of Helsinki and Good Clinical Practice Guidelines and was approved by the Independent Ethics Committee of the coordinating center Hospital Ramón y Cajal (ceic.hrc@salud.madrid.org, approval 146-21l) and the participating centers. All participants provided informed consent before the initiation of the study procedures.

### Study population

We conducted an observational, prospective, single-center study. From July 2022 to July 2023, we systematically invited women undergoing routine cervical cytology consultations at their Primary Care Center or Gynecology department assigned to the project in Madrid, Spain. Participants were excluded if they were under 18 years of age, declined to sign informed consent, had received systemic antibiotics in the previous month, or presented with systemic inflammatory conditions (such as chronic hepatitis B or C, intercurrent infections, or autoimmune diseases), in order to minimize major disturbances to the microbiota. We included 105 women classified into three groups: women who tested negative for HPV and had normal cytology (NEG), those who tested positive for HPV and had low-grade squamous intraepithelial lesions (LSIL), and those who tested positive and had high-grade squamous intraepithelial lesions (HSIL).

All the women provided written consent to participate in the study. Endocervical samples were collected using sterile endocervical brushes (Cervex-Brush® Combi, Rovers Medical Devices) by trained gynecologists or primary care physicians. The brush was gently inserted into the endocervical canal and rotated 360° to maximize epithelial contact while minimizing contamination from the vaginal introitus.

HPV detection was performed using the Cobas® 4,800 HPV Test, which individually detects HPV16 and HPV18, and reports the presence of 12 additional high-risk genotypes (HPV-31, −33, −35, −39, −45, −51, −52, −56, −58, −59, −66, and −68) collectively as “other HR-HPV.” No specific genotyping was performed for these additional genotypes.

### 16S rRNA sequencing analysis

Cryopreserved samples obtained in sterile iSWAB-Microbiome tubes were thawed and DNA was extracted using the QIAamp DNA Mini Kit. For each sample, the V3-V4 regions of the 16S rRNA gene were analyzed using Illumina MiSeq (Novogene Bioinformatics Technology Co., Ltd.). 50 K reads with 250 bp Pair-Ends inserts were analyzed for OTUs cluster and phylogenetic relationship construction. Different Alpha and Beta diversity indexes were calculated using Simpson, Shannon, and Principal Coordinate Analysis (PCoA) respectively. Differential abundance and function prediction analyses were performed by analyzing the compositions of microbiomes with bias correction (ANCOM-BC2) (11) identifying significantly different orthologous genes using the Kyoto Encyclopedia of Genes and Genomes (KEGG/KO) and Enzyme Commission (EC) terms between groups in the dataset. Bioinformatic analysis was performed using the Novogene pipeline. We then selected these terms and used MicrobiomeAnalyst to identify the enriched metabolic pathways or biological processes associated with them.

### Statistical analysis

Study data were collected and managed using REDCap electronic data capture tools v13.1.27. We report qualitative variables as frequency distribution and quantitative variables as medians with their 25th-75th percentiles (P25-P75) or as means and standard deviation (SD), according to the data distribution. We performed comparisons between groups using the Chi-square test for categorical variables. Since the distribution of all the assessed variables deviated from normality, we used the Mann–Whitney U test for between-group comparisons of continuous variables. We used Stata v18.0 (StataCorp LP, College Station, TX, USA) to perform the statistical tests. Beta diversity was primarily assessed using weighted UniFrac distances to account for differences in taxon abundance across groups. Differential abundance of bacterial taxa was assessed using ANCOM-BC2, with statistical significance defined as a false discovery rate (FDR)–adjusted q-value < 0.05. For functional enrichment analysis based on KEGG Orthologs (KO) and Enzyme Commission (EC) terms, we considered pathways with a raw *p*-value < 0.01 as significant.

## Results

### General characteristics

We included 105 women: 87, and 18 in the non-HSIL, and HSIL groups, respectively. The median age of the participants was similar across the groups: 41 years in the non-HSIL group, 38.5 years in the HSIL group. Most participants were Spanish (86.2, and 72.2% of the non-HSIL, and HSIL groups, respectively). A smaller proportion of participants were from Eastern Europe and South America.

We found differences among the groups in terms of health determinants such as smoking and HPV vaccination (see Table 1 and Supplementary Table S1). Women in the HSIL groups had a higher prevalence of current smoking than those in the non-HSIL group did and women in the HSIL group were more frequently in a stable relationship compared to those in the non-HSIL group (78% vs. 46%, *p* = 0.031). Additionally, there were notable differences in HPV vaccination rates, with a higher percentage of vaccinated women in the non-HSIL group than in the HSIL group.

**Table 1:** General characteristics of the study participants dividing the groups in two categories (non-HSIL and HSIL).

Variable		non-HSIL (*N* = 87)	HSIL (*N* = 18)	*p*-value
Age, median (IQR)		41 (34–48)	38.5 (34.5–45.5)	0.9
Nationality, *n* (%)	Spain	75 (86.2%)	13 (72.2%)	0.136
	Eastern Europe	4 (4.6%)	1 (5.5%)	
	South America	6 (6.9%)	4 (22.2%)	
Current smoker, *n* (%)	No	45 (51.7%)	12 (66.7%)	0.874
	Yes	14 (16.1%)	5 (27.8%)	
Condom use, *n* (%)	Never	43 (49.4%)	13 (72.2%)	0.892
	< 50%	2 (2.3%)	0 (0%)	
	> 50%	4 (4.6%)	1 (5.6%)	
	Always	10 (11.5%)	3 (16.7%)	
Currently in a couple, *n* (%)	No:	19 (21.8%)	3 (16.7%)	<0.05
	Yes	40 (46.0%)	14 (77.8%)	
	N/A	28 (32.2%)	1 (5.6%)	
STIs in the last year, *n* (%)	No	84 (96.5%)	17 (94.4%)	1.000
	Yes	2 (2.3%)	1 (5.6%)	
HPV vaccine, *n* (%)	Yes	18 (20.7%)	2 (11.1%)	0.540
	No	69 (79.3%)	16 (88.9%)	
HPV-AR, *n* (%)	HPV16/18	5 (5.7%)	5 (27.7%)	<0.05
	Other HR types	29 (33.3%)	11 (61.1%)	
	None	53 (60.9%)	2 (11.1%)	

STI, Sexually transmitted infections; HPV, Human Papilloma Virus; HSIL, high-grade squamous intraepithelial lesions HR: High Risk.

Values not represented correspond to non-responses from patients. IQR, interquartile range.

Among HPV-seropositive women, the distribution of HPV types was different between the non-HSIL and HSIL groups (*p* < 0.05). Most high-risk HPV genotypes were neither 16 nor 18. For these high-risk HPV genotypes, the specific type was not identified by polymerase chain reaction (PCR), which means that it could be any of the following: 31, 33, 35, 39, 45, 51, 52, 56, 58, 59, 59, 66, and 68. However, the strong enrichment of HPV16/18 in HSIL is consistent with their established role in cervical carcinogenesis, and supports the validity of our dichotomized grouping strategy for microbiota analyses. For more information, please refer to Table 1 and Supplementary Table S1.

### Microbiota diversity and specific taxa associations in the development of cervical HSIL

Changes in microbiota and metabolism have been documented across different stages of CC. We analyzed the differences between the different stages (Negative, LSIL and HSIL) (see Supplementary Figure S1). However, we decided to focus on microbiota analyses by dividing the samples into two general groups: women who developed HSIL (HSIL) and those who did not develop HSIL (non-HSIL) to prioritize a clinically actionable and parsimonious grouping, since only HSIL requires treatment, and to focus on extreme phenotypes, which may help to identify more robust microbial markers.

**Figure 1:**
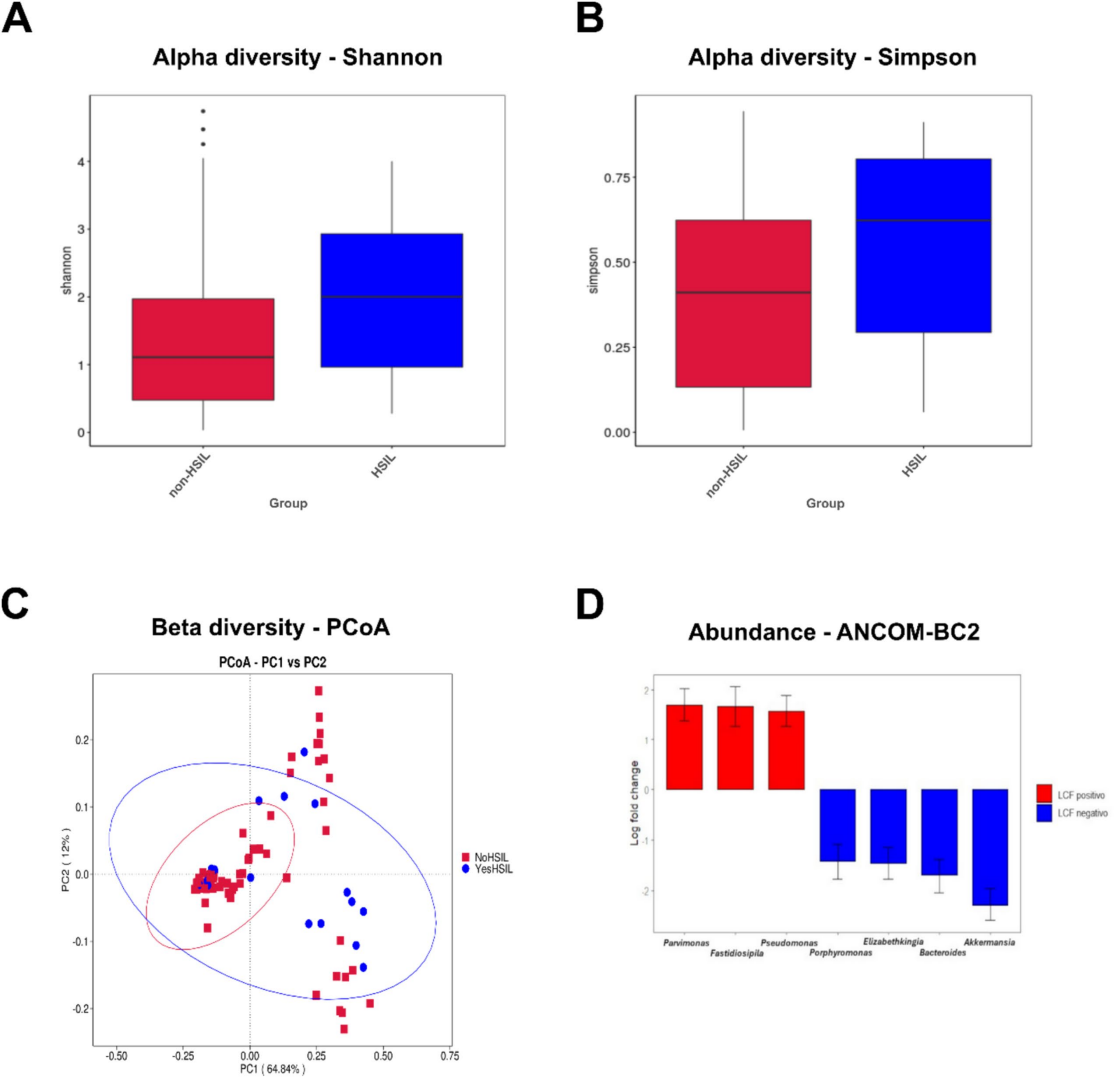
Diversity and abundance of cervicovaginal microbiota in the two compared groups (Non-HSIL and HSIL). **(A)** Boxplots based on Shannon index, showing the maximum, minimum, median, and abnormal values of the index from each group. Wilcoxon two-tailed test *p*-value: 0.036. **(B)** Boxplots based on the Simpson index, showing the maximum, minimum, median, and abnormal values of the index from each group. Wilcoxon two-tailed test *p*-value: 0.024. **(C)** PCoA based on the weighted Unifrac distance showing the two components explaining most of the variance (PC1 and PC2). The multiresponse permutation procedure (MRPP) test was performed to statistically evaluate the differences between the groups (MRPP significance: non-HSIL-HSIL = 0.238). **(D)** The ANCOM-BC2 test was performed to analyze the abundance of genera in the two groups. The results were calculated as Log Fold Change (LFC). Red indicates genera with a positive LFC (higher abundance) in the HSIL group. Blue indicates genera with a negative LFC (lower abundance) in the HSIL group.

To measure the richness and evenness of bacterial taxa within a community, we calculated alpha diversity indexes (Figures 1A,B). Alpha diversity, calculated using Shannon and Simpson indexes, varied significantly between the HSIL and non-HSIL groups. We also applied a dimensionality-reduction technique, Principal Coordinates Analysis (PCoA), to visualize the differences between microbial community configurations (beta diversity), and we found no clearly distinct clusters (Figure 1C). To identify specific taxa that differed significantly in abundance between groups, we used the ANCOM-BC2 method to compare the differences in absolute abundances. Women with HSIL showed increased abundances in the genus *Parvimonas, Fastidiosipila,* and *Pseudomonas*, while the genera *Porphyromonas*, *Elizabethkingia*, *Bacteroides,* and *Akkermansia* were depleted (Figure 1D).

### Functional analysis of the cervical microbiome reveals key pathways associated with the development of cervical HSIL

**Figure 2:**
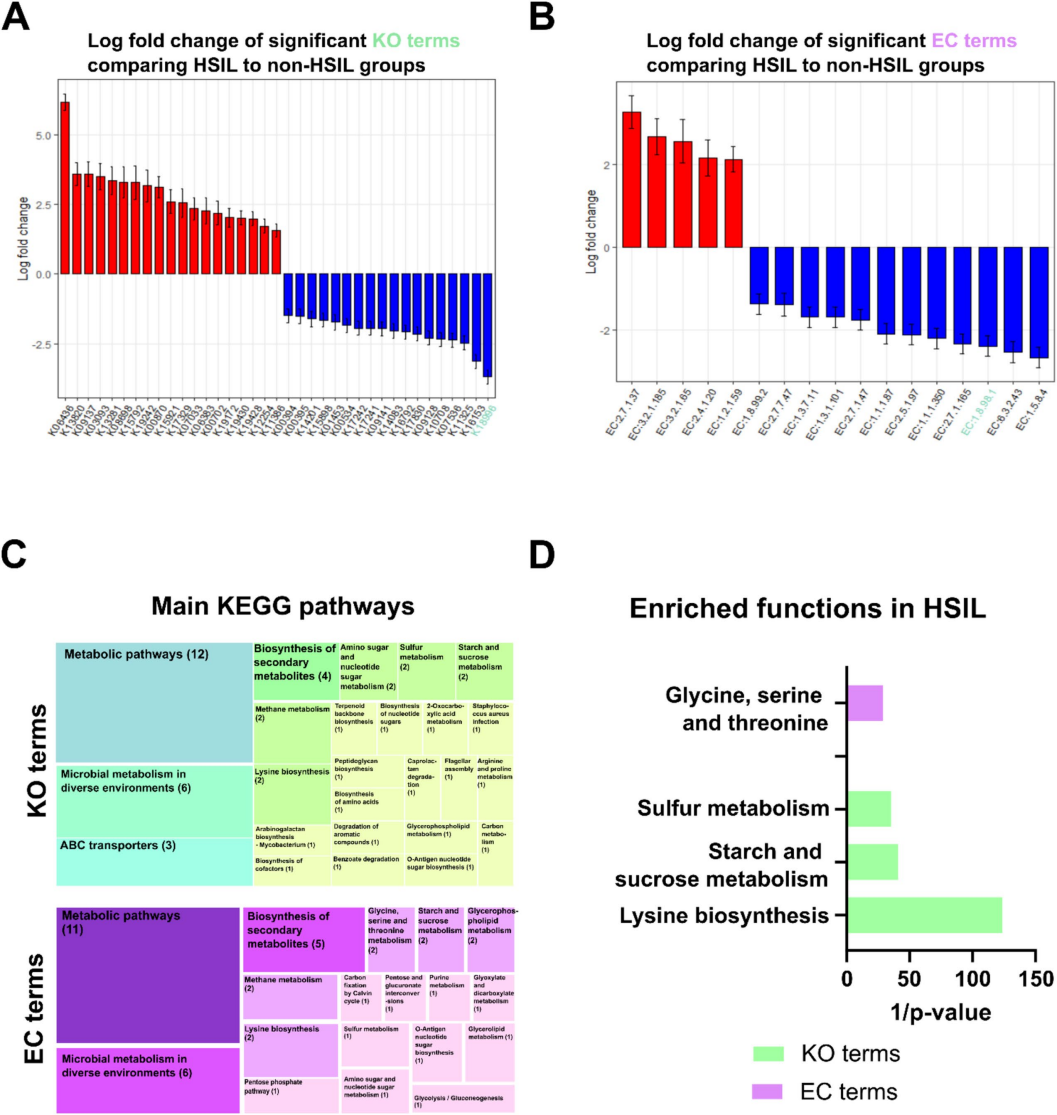
Functional terms differentially found in the cervical HSIL group compared to the negative group. **(A)** KEGG terms obtained using ANCOM-BC2 and selected based on their LFC. **(B)** EC terms obtained using ANCOM-BC2 and selected based on their LFC. The genus in red had a positive LFC (higher abundance) in the group with HSIL. The genus in blue had a negative LFC (lower abundance) in the HSIL group. Genus subjected to pseudocount correction by ANCOM-BC2 is shown in green. **(C)** The main terms were obtained using KEGG mapper with the list of significant terms from the ANCOM-BC2 analysis. Numbers in parentheses denote the number of terms in each category. **(D)** Main terms obtained after performing enrichment analysis, as explained in the Methods section, for the KO terms (green) and EC terms (purple) obtained using ANCOM-BC2. The inverse of the *p*-value is represented on the x-axis for visualization purposes.

Next, we sought to understand the potential mechanisms by which the cervical microbiome influences the development of HSIL. We compared the bacterial imputed functions between the most extreme phenotypes, the non-HSIL and HSIL groups. We also applied the ANCOM-BC2 method, focusing on the KEGG and EC databases, to identify the most significant terms. We identified 37 KO and 17 EC terms that were significantly associated with HSIL (Figure 2; Supplementary Tables S2, S3).

This two-step approach allows us to first identify relevant genes through differential abundance.

Analysis and interpretation of the functional impact of these genes were performed by mapping them to pathways, reducing noise, and increasing the biological relevance of our results. In women with HSIL, several KO terms related to metabolic pathways, biosynthesis of secondary metabolites, and microbial metabolism were significantly enriched, including those associated with amino sugar and nucleotide metabolism, sulfur metabolism, and starch/sucrose metabolism. On the enzyme side, key EC terms related to lysine biosynthesis, carbohydrate degradation, and glyoxylate metabolism were more abundant, whereas pathways such as glycine/serine/threonine metabolism were depleted.

## Discussion

Our study revealed a more diverse vaginal microbiota in women with cervical dysplasia than that in healthy women, with differences in specific taxa and biological functions linked to the development of cervical HSIL. Increased microbial diversity in the cervical environment, as observed in women with HSIL, may reflect a shift away from a Lactobacillus-dominated state toward a polymicrobial community enriched in anaerobes and associated with local inflammation. Such transitions have been linked to epithelial barrier disruption, altered immune responses, and HPV persistence, all of which may contribute to lesion progression (12, 13). The increased abundance of *Parvimonas, Fastidiosipila*, and *Pseudomonas* in women with HSIL suggests their potential role in cervical dysbiosis and HPV progression. Notably, *Parvimonas* spp. has been frequently associated with precancerous lesions and dysbiosis (7), particularly in connection with sexually transmitted infections such as *Chlamydia trachomatis* and persistent HPV infection. Interestingly, *Pseudomonas,* while often considered part of the healthy endocervical microbiota, has been linked to various pathologies such as *Chlamydia trachomatis* infection in some *in vitro* studies (14, 15), further supporting the idea that shifts in the microbiota may contribute to disease. The prominence of these genera, which are more commonly associated with the intestinal microbiota, suggests possible translocation or shared pathways between the gut and the cervicovaginal environment (16, 17). Mechanisms such as bacterial ascent through the reproductive tract or hematogenous spread from gastrointestinal niches could explain this crossover. However, further studies are required to explore these possibilities.

The functional analysis further reinforces the association between microbial dysbiosis and HSIL. We found enriched gene pathways related to typical bacterial metabolic processes and enzyme pathways related to glycine, serine, and threonine metabolism, suggesting that the microbial community in HSIL cases exhibits heightened metabolic activity. The increase in glycosidases and a corresponding decrease in oxidoreductases also point to significant metabolic alterations, potentially contributing to a dysbiotic environment conducive to lesion progression. These findings are consistent with metabolic dysregulation linked to cancer development, including processes such as the Warburg effect and oxidative stress, which may promote DNA damage and promote the development of cancerous lesions (18, 19).

Our study has some limitations. First, although we observed robust and consistent associations across diversity indices, taxonomic composition, and functional pathways, the relatively small number of HSIL cases (*n* = 18) and the amplicon-based sequencing technique may have limited our ability to detect more subtle differences. Nonetheless, the effect sizes observed were sufficient to reach statistical significance despite this limitation. Second, certain behavioral and hormonal factors known to influence the cervicovaginal microbiota—such as contraceptive use, recent antibiotic exposure, or sexual behavior—were not systematically collected, which may represent sources of residual confounding. While our findings are representative of a well-defined Spanish population, we acknowledge the need for larger, multicenter, and multiethnic studies to confirm and extend these results. Importantly, the microbial shifts we observed are consistent with reports from other settings, supporting the biological plausibility and relevance of our findings. Although our study was not designed to assess the clinical utility of microbiota-based markers, the microbial and functional differences observed may help inform future efforts to develop adjunctive tools for risk stratification. We have previously shown the predictive value of microbiome and metabolomic signatures for anal HSIL progression in a prospective cohort (20), and similar strategies are now being planned for cervical dysplasia.

Microbiome-based interventions, particularly intravaginal administration of *Lactobacillus crispatus* and long-term use of *Lactobacillus rhamnosus*, have demonstrated potential in improving cervical cytology and promoting high-risk HPV clearance in small randomized or controlled trials (20, 21). While these findings are promising, they remain limited by small sample sizes and short follow-up durations. Larger, rigorously designed randomized controlled trials are urgently needed to confirm efficacy and guide clinical implementation. Additionally, most studies examining the relationship between microbiota and cervical dysplasia have focused on vaginal rather than cervical samples (21) or have reported mixed results from different anatomical sites (22). Future studies correlating both environments could provide a less invasive means of assessing women at a higher risk of progressing to HSIL by linking findings in the vaginal microbiota to those in the cervix.

Geographic location may also influence microbiota composition (23). To the best of our knowledge, this study is the first to examine the link between the cervical microbiota and HSIL in women from Spain, and direct comparisons with the existing literature are challenging. This underscores the need for region-specific studies to better understand the microbial landscape and its relationship to cervical dysplasia. Lastly, although our functional analysis identified significant pathways, the presence of genes does not guarantee their activity, highlighting the importance of further experimental studies, particularly longitudinal studies, to establish causal relationships between cervical dysbiosis and CIN progression.

We acknowledge that cross-sectional studies such as ours cannot determine causality. Although key exclusion criteria were applied to reduce major confounders, we acknowledge that many other factors influencing microbiota composition—such as diet, probiotic use, or menstrual cycle phase—were not systematically collected and represent a limitation of this study. In our regrouped analysis (HSIL vs. non-HSIL), being currently in a couple was more frequent among women with HSIL, however, while this finding may reflect unmeasured aspects of sexual behavior or partner-related factors, it should be interpreted cautiously given the lack of detailed behavioral data. These limitations have been broadly discussed in our recent review (24), where we emphasized the need for complementary experimental models —such as experimental models, multi-omics approaches, and longitudinal cohort designs—to better elucidate the causal relationships between the microbiota and viral pathogenesis. Although we observed differences in variables such as smoking, age, or nationality across groups, our study was not designed or powered to disentangle their individual effects on microbiota composition. Future hypothesis-driven studies with larger sample sizes and multivariable modeling will be required to evaluate the independent contribution of these host factors.

Despite these limitations, our study lays the groundwork for future research to explore the role of the cervicovaginal microbiome in HSIL development. The microbial taxa and metabolic pathways identified here could inform studies focused on identifying markers for cervical dysplasia progression, offering avenues for targeted screening and therapeutic interventions.

## Data Availability

The datasets presented in this study can be found in online repositories. The names of the repository/repositories and accession number(s) can be found in the article/Supplementary material.
