# Supplementary material for: Cervical microbiota diversity and functional shifts in the development of cervical high-grade squamous intraepithelial lesions
Source: Front Med (Lausanne). 2025 Oct 24;12:1615571. doi: 10.3389/fmed.2025.1615571 (PMC12592144; doi:10.3389/fmed.2025.1615571)
Supplement: Supplementary file 1 [file Supplementary_file_1.docx]

**Supplementary Material for**

**Cervical Microbiota Diversity and Functional Shifts in the Development of Cervical High-Grade Squamous Intraepithelial Lesions**

Marta Rosas Cancio-Suárez^1,2,5^ *, Elena Moreno^1,2^ *, Cristina del Valle Rubido^3^, Marta Salvador^3^, Ana I. Moreno Gómez^4^, Laura Luna^1,2^, Claudio Díaz-García^,1,2^, Carlos Tapia^1^, Ana del Amo^1^, Santiago Moreno^1,2,5^, Matilde Sánchez-Conde^,1,2^, Sergio Serrano-Villar^1,2^

*These authors contributed equally

**Affiliations:**

1. Department of Infectious Diseases, IRYCIS, and Universidad de Alcalá, Hospital Universitario Ramón y Cajal, 28034, Madrid, Spain
2. CIBERINFEC, Instituto de Salud Carlos III, Madrid, Spain
3. Department of Ginecology, IRYCIS, Hospital Universitario Ramón y Cajal, 28034, Madrid, Spain
4. Alpes Primary Care Center, 28022 Madrid, Spain.
5. Department of Medicine, University of Alcalá de Henares, Guadalajara Campus, 28801 Alcalá de Henares, Spain

**Corresponding author**: [marta.rosas@salud.madrid.org](mailto:marta.rosas@salud.madrid.org) (M.R.C-S.) and emolmo@salud.madrid.org (E.M.).

**Figure S1. Diversity and abundance of cervicovaginal microbiota comparing the three groups according stages of HPV-associated cervical transformation.**

**
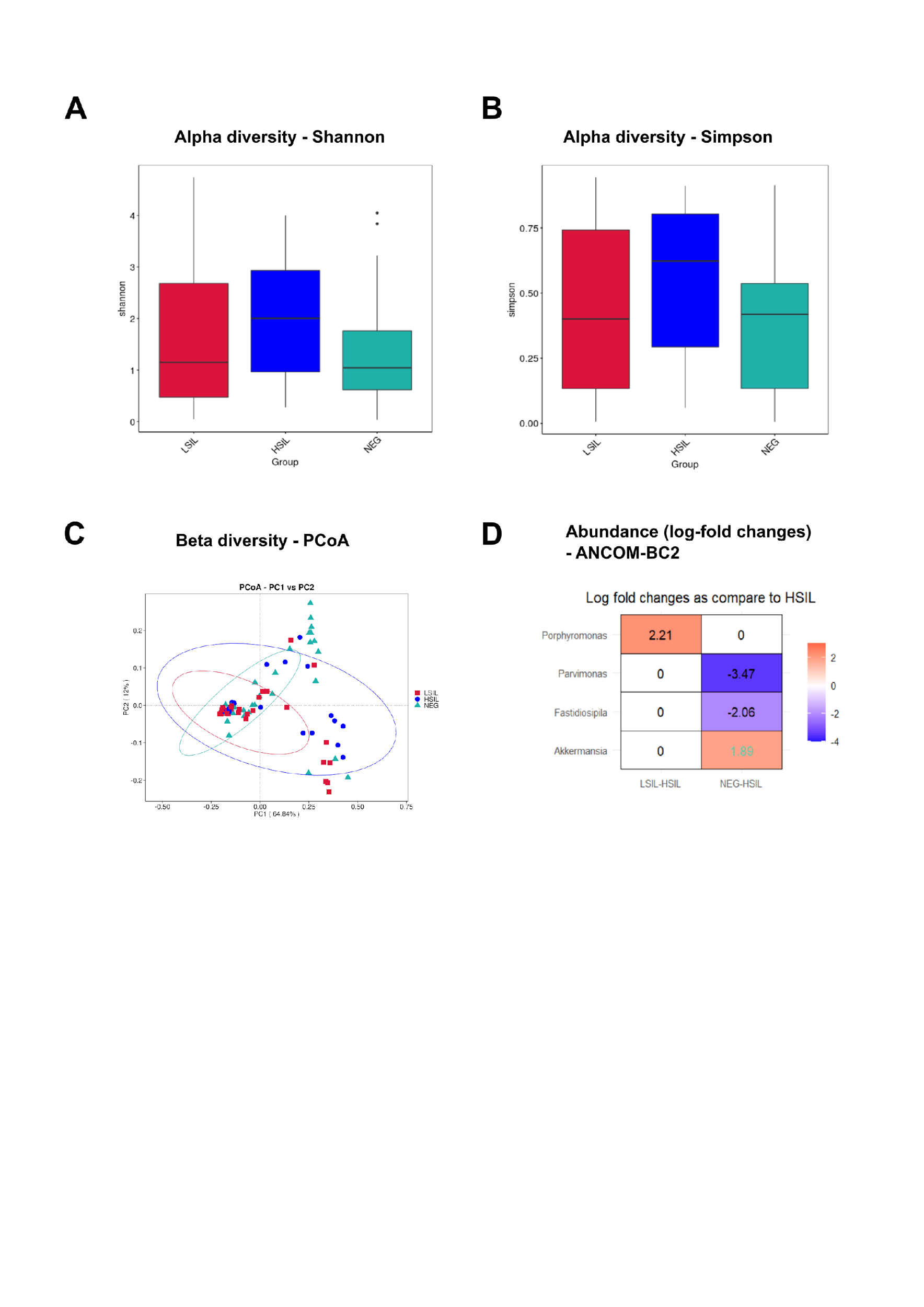
**

**Figure S1. A**. Boxplots based on Shannon index, showing the maximum, minimum, median and abnormal values of the index from each group. Kruskal Wallis test p-values: HSIL vs LSIL = 0.12, HSIL vs NEG = 0.03, LSIL vs NEG = 0.50. **B.** Boxplots based on Simpson index, showing the maximum, minimum, median and abnormal values of the index from each group. Kruskal Wallis test p-values: HSIL vs LSIL = 0.09, HSIL vs NEG = 0.02, LSIL vs NEG = 0.48. **C.** PCoA based on the weighted Unifrac distance showing the two components explaining most of the variance (PC1 and PC2). The multiresponse permutation procedure (MRPP) test was performed to statistically evaluate the differences between the groups (MRPP significance: HSIL vs LSIL = 0.162, HSIL vs NEG = 0.342, LSIL vs NEG = 0.298). **D.** The ANCOM-BC2 test was performed to analyze the abundance of genera in the three groups. The results were calculated as Log Fold Change (LFC). Red indicates genera with a positive LFC (higher abundance) in the HSIL group. Blue indicates genera with a negative LFC (lower abundance) in the HSIL group. Taxa names highlighted in green passed ANCOM-BC2’s pseudo-count sensitivity test, meaning their differential abundance findings are robust to zero-count handling.

**Table S1. General characteristics of the study participants dividing the groups in three categories (Negative, LSIL and HSIL).**

|  |  | **Negative HPV and normal cytology (N=53)** | **LSIL**  **(N=34)** | **HSIL (N=18)** | **p-value** |
| --- | --- | --- | --- | --- | --- |
| **Age, median (IQR)** |  | 42 (34-48) | 41 (36.2-44.7) | 38.5 (34.5-45.5) | 0.80 |
| **Nationality, n (%)** | Spain (N=88) | 50 (94.3%) | 25 (73.5%) | 13 (72.2%) | 0.07 |
|  | East Europe (N=5) | 1 (1.9%) | 3 (8.8%) | 1 (5.5%) |  |
|  | South America (N=10) | 2 (5.9%) | 4 (11.7%) | 4 (22.2%) |  |
| **Current smoker, n (%)** | No | 22 (41.5) | 23 (67.6%) | 12 (66.7%) | <0.05 |
|  | Yes | 4 (7.5%) | 10 (29.4%) | 5 (27.8%) |  |
| **Condom use, n (%)** | Never | 24 (45.3%) | 19 (55.9%) | 13 (72.2%) | 0.1 |
|  | < 50% | 1 (1.9%) | 1 (2.9%) |  |  |
|  | > 50% | 0 (0%) | 4 (5.9%) | 1 (5.6%) |  |
|  | Always | 2 (3.8%) | 8 (23.5%) | 3 (16.7%) |  |
| **Currently in a couple, n (%)** | No | 5 (9.4%) | 14 (41.2%) | 3 (16.7%) | <0.05 |
|  | Yes | 22 (41.5) | 18 (52.9%) | 14 (77.8%) |  |
|  | N/A | 26 (49.1%) | 2 (5.9%) | 1 (5.6%) |  |
| **STIs in the last year, n (%)** | No | 52 (98.1%) | 32 (94.1%) | 17 (94.4%) | 0.72 |
|  | Yes | 1 (1.9%) | 1 (2.9%) | 1 (5.6%) |  |
| **HPV vaccine, n (%)** |  | 10 (18.9%) | 8 (23.5%) | 2 (11.1%) | <0.05 |
| **HPV-AR, n (%)** | 16 or 18 | 0 (0%) | 5 (14,7%) | 5 (27.7%) | <0.05 |
|  | No 16 nor 18 | 0 (0%) | 29 (85.3%) | 11 (61.1%) |  |
|  | None | 53 (100%) | 0 (0%) | 2 (11.1%) |  |
| STI: Sexually transmitted infections; HPV: Human Papilloma Virus; LSIL: low-grade squamous intraepithelial lesions HSIL: high-grade squamous intraepithelial lesions HR: High Risk.  Note: Values not represented correspond to non-responses from patients. IQR, interquartile range | | | | | |

**Table S2: LCF of KO comparing samples from women without lesions to HSIL samples.**

This table shows the KO terms with a log fold change (LCF) greater than 3 when comparing women without lesions to the HSIL sample.

| KO | Description | LCF | SE | P | P ajusted | |
| --- | --- | --- | --- | --- | --- | --- |
| K06436 | yabG; spore coat assembly protein | 6.16 | 0.30 | 7.25E-16 | | 2.00E-12 |
| K13820 | fliR-flhB; flagellar biosynthetic protein FliR/FlhB | 3.59 | 0.41 | 1.12E-08 | | 3.09E-05 |
| K09137 | Uncharacterized | 3.59 | 0.44 | 1.05E-08 | | 2.89E-05 |
| K03093 | sigI; RNA polymerase sigma factor | 3.49 | 0.47 | 4.82E-08 | | 1.33E-04 |
| K13281 | uvsE; UVE1; UV damage DNA endonuclease | 3.35 | 0.49 | 1.72E-06 | | 4.75E-03 |
| K08698 | ccmM; carbon dioxide concentration mechanism protein | 3.30 | 0.55 | 3.37E-06 | | 9.26E-03 |
| K15792 | murEF; murE/murF fusion protein | 3.28 | 0.61 | 1.81E-06 | | 4.99E-03 |
| K19242 | citR; LysR family transcriptional regulator, citA repressor | 3.16 | 0.56 | 2.82E-06 | | 7.74E-03 |
| K16153 | K16153; glycogen phosphorylase/synthase | -3.13 | 0.25 | 2.43E-09 | | 6.72E-06 |
| K18996 | repC; replication initiation protein RepC | -3.69 | 0.25 | 6.46E-12 | | 1.79E-08 |

LCF: *Log Fold Change for HSIL. SE: Standard Error of the change for HSIL. P: P-value. Adjusted P: P-value adjusted for FDR (False Discovery Rate). P-values are expressed in scientific notation.*

**Table S3: LCF of EC comparing samples from women without lesions to HSIL samples.**

This table shows the EC terms with a log-fold change (LCF) greater than 3 when comparing women without lesions to the HSIL sample.

| **EC** | **LCF** | **SE** | **P** | **P ajusted** |
| --- | --- | --- | --- | --- |
| EC:1.5.8.4 | 3.27 | 0.25 | 9.15E-08 | 9.56E-05 |
| EC:6.3.2.43 | 2.67 | 0.25 | 9.71E-08 | 1.01E-04 |
| EC:1.8.98.1 | 2.56 | 0.25 | 9.91E-10 | 1.04E-06 |
| EC:1.2.1.59 | -2.09 | 0.30 | 9.12E-08 | 9.54E-05 |
| EC:2.4.1.20 | -2.11 | 0.44 | 1.93E-05 | 2.00E-02 |
| EC:1.3.8.1 | -2.21 | 0.64 | 8.67E-04 | 8.78E-01 |
| EC:3.2.1.187 | -2.34 | 0.44 | 1.60E-04 | 1.64E-01 |
| EC:3.2.1.65 | -2.39 | 0.52 | 7.48E-06 | 7.78E-03 |
| EC:3.2.1.185 | -2.53 | 0.44 | 2.32E-05 | 2.40E-02 |
| EC:2.7.1.37 | -2.67 | 0.39 | 2.16E-09 | 2.26E-06 |

LCF: *Log Fold Change for HSIL. SE: Standard Error of the change for HSIL. P: P-value. Adjusted P: P-value adjusted for FDR (False Discovery Rate). P-values are expressed in scientific notation.*
